# Mosquito Control Based on Pesticides and Endosymbiotic Bacterium *Wolbachia*

**DOI:** 10.1007/s11538-021-00881-9

**Published:** 2021-04-13

**Authors:** Linchao Hu, Cui Yang, Yuanxian Hui, Jianshe Yu

**Affiliations:** 1 Center for Applied Mathematics, Guangzhou University, Guangzhou, 510006 People’s Republic of China; 2 College of Mathematics and Information Sciences, Guangzhou University, Guangzhou, 510006 People’s Republic of China; 3 The Second Affiliated Hospital of Guangzhou Medical University, Guangzhou, 510260 People’s Republic of China

**Keywords:** *Wolbachia*, Mosquito-borne diseases, Population replacement, Population suppression, Pesticides, Heatwave

## Abstract

Mosquito-borne diseases, such as dengue fever and Zika, have posed a serious threat to human health around the world. Controlling vector mosquitoes is an effective method to prevent these diseases. Spraying pesticides has been the main approach of reducing mosquito population, but it is not a sustainable solution due to the growing insecticide resistance. One promising complementary method is the release of *Wolbachia*-infected mosquitoes into wild mosquito populations, which has been proven to be a novel and environment-friendly way for mosquito control. In this paper, we incorporate consideration of releasing infected sterile mosquitoes and spraying pesticides to aim to reduce wild mosquito populations based on the population replacement model. We present the estimations for the number of wild mosquitoes or infection density in a normal environment and then discuss how to offset the effect of the heatwave, which can cause infected mosquitoes to lose *Wolbachia* infection. Finally, we give the waiting time to suppress wild mosquito population to a given threshold size by numerical simulations.

## Introduction

Mosquito-borne diseases, such as dengue fever and Zika, have posed a serious threat to the health of human beings around the world and bring a great financial burden to the governments in the tropic and sub-tropic areas (Kyle and Harris 2008; Rasmussen et al. 2016). Since there are no efficient vaccines available, controlling the vector population is the most effective measure of preventing mosquito-borne diseases. For a long time, vector control methods have mainly relied on the extensive use of insecticides. Although the utilization of insecticides reduces the mosquito population size greatly, it causes environmental pollution and offers only a short-term solution due to the mosquito resistance to insecticides (Kyle and Harris 2008; Ooi et al. 2006). An innovative and environmentally friendly strategy for the control of mosquito-borne diseases is to employ the maternally inherited endosymbiotic bacterium *Wolbachia*, whose infection in *Aedes* mosquitoes can reduce their transmission potential to spread viruses (Bian et al. 2010; Dutra et al. 2016). In addition, *Wolbachia* induces cytoplasmic incompatibility (CI) that causes early embryonic death when *Wolbachia*-infected males mate with uninfected females (Laven 1956), resulting in the decrease of the proportion of uninfected mosquitoes. Therefore, we can release infected mosquitoes to invade and replace the wild population (population replacement) or suppress wild mosquito population to reduce mosquito bites (population suppression). There has been an increasing interest toward the spreading dynamics of *Wolbachia*; see (Farkas and Hinow 2010; Hu et al. 2019; Keeling et al. 2003; Shi and Yu 2020; Yu and Zheng 2019) for the theoretical works on population replacement and (Huang et al. 2018, 2020; Yu 2018; Zhang et al. 2020) on population suppression. By releasing infected mosquitoes twice a week, our team, led by Xi, eradicated more than 90% of *Aedes albopictus* in an island in South Guangzhou (Zheng et al. 2019), which verifies the feasibility of mosquito suppression in the field. On the other hand, it is reported in Nature News that releasing *Wolbachia-*infected mosquitoes in Yogyakarta reduces 77% of dengue cases compared with areas that did not receive infected mosquitoes (Callaway 2020). The regression model in Ryan et al. (2019) also showed a 96% reduction in dengue incidence in *Wolbachia*-treated populations. These trials proved that population replacement based on *Wolbachia* may greatly block the transmission of mosquito-borne diseases.

Suggested by the empirical data (McMeniman et al. 2009; Walker et al. 2011; Yeap et al. 2011), we give three basic assumptions: perfect maternal transmission, complete CI, and equal sex determination. Motivated by the work in Yu (2018), let $b_I$ and $b_U$ be the total numbers of offspring per unit of time, per infected and uninfected mosquitoes, respectively. Let $\delta _I$ and $\delta _U$ denote the density-independent decay rates of infected and uninfected mosquitoes, and $d_I$ and $d_U$ the density-dependent decay rates of infected and uninfected mosquitoes, respectively. Denote by *x*(*t*) and *y*(*t*) the numbers of infected and uninfected mosquitoes, respectively. Then we obtain the following differential equation model to characterize the dynamics of infected and uninfected mosquitoes,

1 $$\begin{aligned} \left\{ \begin{array}{ll} \displaystyle \frac{\mathrm{d}x}{\mathrm{d}t}=(b_I-\delta _I)x-d_Ix(x+y),\\ \displaystyle \frac{\mathrm{d}y}{\mathrm{d}t}=b_Uy\frac{y}{x+y}-\delta _Uy-d_Uy(x+y). \end{array} \right. \end{aligned}$$

The term $y/(x+y)$ represents is the probability of mating with wild mosquitoes. Since the infected or uninfected mosquitoes don’t die out naturally in the wild, we assume

2 $$\begin{aligned} b_I>d_I+\delta _I,~~~~~b_U>d_U+\delta _U. \end{aligned}$$

In general, *Wolbachia* infections bring fitness cost to their hosts such as reduced fecundity or longevity (McMeniman et al. 2009; Walker et al. 2011; Weeks et al. 2002). Here we consider these differences and ignore the diversity of density-dependent death rates between infected and uninfected mosquitoes based on Zhang et al. (2015). Then we assume that

3 $$\begin{aligned} b_U\ge b_I,~~~~~\delta _I\ge \delta _U~~~~~\text {and}~~~~~d_I=d_U=d. \end{aligned}$$

Most of the existing literatures discuss population replacement or population repression separately. In this work, we consider subsequent release of infected sterile mosquitoes and spraying insecticides based on the population replacement model (1). Let *R*(*t*) be the release abundance of infected mosquitoes at time *t*. Let $\phi _I(t)$ and $\phi _U(t)$ denote the excess death rates caused by pesticides for infected and uninfected mosquitoes, respectively. Then we obtain the following model by combining the use of pesticides and the release of infected mosquitoes:

4 $$\begin{aligned} \left\{ \begin{array}{ll} \displaystyle \frac{\mathrm{d}x}{\mathrm{d}t}=(b_I-\delta _I-\phi _I(t))x-dx(x+y),\\ \displaystyle \frac{\mathrm{d}y}{\mathrm{d}t}=b_Uy\frac{y}{x+y+R(t)}-(\delta _U+\phi _U(t))y-dy(x+y). \end{array} \right. \end{aligned}$$

Recently, scientists found that infected mosquitoes may lose *Wolbachia* at egg and larvae stages due to the strike of heatwaves (Ross et al. 2020, 2017). This leakage situation has been discussed in (Farkas and Hinow 2010; Keeling et al. 2003; Zheng et al. 2018). Let $\mu$ denote the fraction of uninfected offspring produced by infected mosquitoes. By reconsidering the numbers of new born offspring of both uninfected and infected mosquitoes based on (4), we obtain an improved model in Sect. 3.

Some mosquito-borne diseases occur periodically and are triggered by imported patients, for example the dengue fever in Guangzhou. It requires emergency measures when the dengue cases in the neighboring areas are large. The empirical data in Guangzhou show that the wild mosquito population must be reduced to a low level such that the Breteau index is less that 5 to prevent dengue fever (Duan et al. 2009). Then we can estimate a safe threshold number ${\mathcal {S}}$ of wild mosquitoes as suppression goal. In Hu et al. (2015), Hu et al. (2019), we discussed the sufficient conditions for *Wolbachia* fixation in deterministic or stochastic environment. In this study, we continue to investigate the detailed dynamical behavior of the wild mosquitoes and estimate the time required (waiting time) to reduce wild mosquitoes to a level below ${\mathcal {S}}$. With the help of numerical simulations, we see that the combination of population replacement and suppression can greatly improve the control speed of wild mosquitoes.

In this work, we start in Sect. 2 with an ordinary differential equation model for mosquito population replacement. By introducing suppression measures, we can accelerate the reduction speed of wild population and we present estimations of the wild mosquito abundance or infection density defined by $p(t)=x(t)/(x(t)+y(t))$. In Sect. 3, we consider a special environmental condition in which the infected mosquitoes may lay uninfected eggs due to the heatwaves. We discuss how to offset this negative effect by releasing infected sterile mosquitoes or spraying pesticides. Finally, we discuss the waiting time to reduce wild mosquitoes to a level below a given threshold in Sect. 4, and shows the requirement for release abundance or spraying density of pesticides for given parameters and initial state.

## Mosquito Control in Normal Environment

**Fig. 1:**
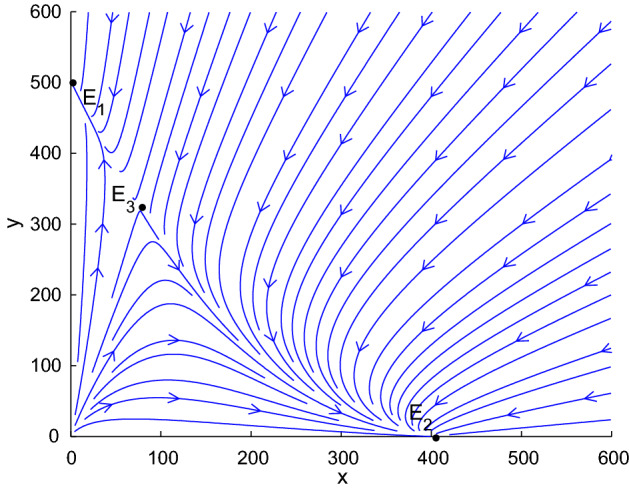
*The vector field direction of system* (1). Let $b_I=0.45$, $b_U=0.55$, $\delta _I=0.05$, $\delta _U=0.048$, $d=0.001$. System (1) admits three equilibria: $E_1$ and $E_2$ are local stable, and they stay on the *y*-axis and *x*-axis, respectively; $E_3$ is a saddle point in the first quadrant

In this part, we introduce two control measures and their combination based on population replacement model (1). According to the assumptions (2) and (3), system (1) admits three equilibria (See Fig. 1): two locally stable equilibria $E_1\left( 0,\frac{b_U-\delta _U}{d}\right) ,~E_2\left( \frac{b_I-\delta _I}{d},0\right) ,$ and a saddle point

$$\begin{aligned} E_3\left( \frac{(b_U-\delta _U-b_I+\delta _I)(b_I-\delta _I)}{b_Ud}, \frac{(\delta _U+b_I-\delta _I)(b_I-\delta _I)}{b_Ud}\right) . \end{aligned}$$

The dynamic behaviors of (1) are similar to the cases in (Farkas and Hinow 2010; Keeling et al. 2003; Zheng et al. 2014). $E_1$ and $E_2$ are local stable and stay on the *y*-axis and *x*-axis, respectively. $E_3$ is a saddle point in the first quadrant. There exists a separatrix $\mathcal {H}:y=h(x)$ in the first quadrant below which the number of *Wolbachia*-infected mosquitoes declines to zero and above which the *Wolbachia*-infected mosquitoes spread to the whole population.

Define the infection density by $p(t)=x(t)/(x(t)+y(t))$. The infection density is easier to monitor than the detailed wild mosquito abundance. Many works discussed the existence of the threshold $p^*$ (Caspari and Watson 1959; Hu et al. 2015; Zheng et al. 2014): the initial infection frequency $p_0> p^*$ leads to *Wolbachia* fixation, while $p_0<p^*$ leads to *Wolbachia* extinction. It follows from (1) that

5 $$\begin{aligned} \frac{\mathrm{d}p(t)}{\mathrm{d}t}= & {} \frac{x'y-xy'}{(x+y)^{2}} \nonumber \\= & {} \frac{xy}{(x+y)^{2}}\bigg (b_I-\delta _I+\delta _U-b_U\frac{y}{x+y}\bigg )\nonumber \\= & {} p(t)(1-p(t))\bigg (b_I-\delta _I+\delta _U-b_U(1-p)\bigg )\nonumber \\= & {} b_Up(t)(1-p(t)) \left( p(t)-\left( 1-\frac{b_I-\delta _I+\delta _U}{b_U}\right) \right) . \end{aligned}$$

Clearly, *p*(*t*) increases in *t* and approaches 1 if $1-\frac{b_I-\delta _I+\delta _U}{b_U}<p(0)<1$, decreases in *t* and approaches 0 if $0<p(0)<1-\frac{b_I-\delta _I+\delta _U}{b_U}$. Thus $1-\frac{b_I-\delta _I+\delta _U}{b_U}$ is a threshold value for the initial infection density above which the *Wolbachia* will invade to the mosquito population successfully, and below which *Wolbachia* frequency declines to zero. Note that $p(t)=1-(b_I-\delta _I+\delta _U)/b_U$ implies $y(t)/x(t)=(b_I+\delta _U-\delta _I)/(b_U-b_I+\delta _I-\delta _U)$. Then

6 $$\begin{aligned} \mathcal {H}:y=h(x)=\frac{b_I+\delta _U-\delta _I}{b_U-b_I+\delta _I-\delta _U}\,x \end{aligned}$$

is the separatrix which divides the first quadrant into two parts, the upper one is the basin of attraction of $E_1$ and the lower one is the basin of attraction of $E_2$. Providing that the initial release ensures successful invasion of *Wolbachia*-infected mosquitoes, we focus on how to reduce wild mosquitoes to a safe level within a given time.

### Repeated Release of Infected Sterile Mosquitoes.

Here we consider repeated release of infected sterile mosquitoes based on population replacement model (1). Then (4) is reduced to

7 $$\begin{aligned} \left\{ \begin{array}{ll} \displaystyle \frac{\mathrm{d}x}{\mathrm{d}t}=(b_I-\delta _I)x-dx(x+y),\\ \displaystyle \frac{\mathrm{d}y}{\mathrm{d}t}=b_Uy\frac{y}{x+y+R(t)}-\delta _Uy-dy(x+y). \end{array} \right. \end{aligned}$$

We first consider the ratio of released mosquito abundance to wild mosquito abundance. Define the release ratio

$$\begin{aligned} {\mathcal {K}}(t):=R(t)/(x(t)+y(t)). \end{aligned}$$

${\mathcal {K}}(t)=0$ corresponding to the case $R(t)=0$. As it is difficult to fix the release ratio to a constant, we let ${\mathcal {K}}_1$ and ${\mathcal {K}}_2$ be the lower and upper bound of ${\mathcal {K}}(t)$, respectively, i.e.,

8 $$\begin{aligned} {\mathcal {K}}_1< {\mathcal {K}}(t) < {\mathcal {K}}_2. \end{aligned}$$

Since $\frac{y}{x+y+R(t)}=\frac{1}{1+{\mathcal {K}}(t)}\cdot \frac{y}{x+y}$, we rewrite (7) as

9 $$\begin{aligned} \left\{ \begin{array}{ll} \displaystyle \frac{\mathrm{d}x}{\mathrm{d}t}=(b_I-\delta _I)x-dx(x+y),\\ \displaystyle \frac{\mathrm{d}y}{\mathrm{d}t}=b_Uy\cdot \frac{1}{1+{\mathcal {K}}(t)}\cdot \frac{y}{x+y}-\delta _Uy-dy(x+y). \end{array} \right. \end{aligned}$$

Define

10 $$\begin{aligned} {\bar{b}}_U=b_U/(1+{\mathcal {K}}_2)~~~\text {and}~~~ {\hat{b}}_U=b_U/(1+{\mathcal {K}}_1). \end{aligned}$$

It follows from (8) that ${\bar{b}}_U< b_U/(1+{\mathcal {K}}(t)) < \hat{b}_U$. We next compare system (9) with the following two systems:

11 $$\begin{aligned}&\left\{ \begin{array}{ll} \displaystyle \frac{\mathrm{d}x}{\mathrm{d}t}=(b_I-\delta _I)x-dx(x+y),\\ \displaystyle \frac{\mathrm{d}y}{\mathrm{d}t}=\bar{b}_Uy\frac{y}{x+y}-\delta _Uy-dy(x+y). \end{array} \right. \end{aligned}$$

12 $$\begin{aligned}&\left\{ \begin{array}{ll} \displaystyle \frac{\mathrm{d}x}{\mathrm{d}t}=(b_I-\delta _I)x-dx(x+y),\\ \displaystyle \frac{\mathrm{d}y}{\mathrm{d}t}=\hat{b}_Uy\frac{y}{x+y}-\delta _Uy-dy(x+y). \end{array} \right. \end{aligned}$$

#### Theorem 1

Let $(x_0,y_0)$ be an initial state with which the solution of (1) approaches $E_2$. Assume $\bar{b}_U<\hat{b}_U$. Let (*X*(*t*), *Y*(*t*)), $(X_1(t),Y_1(t))$ and $(X_2(t),Y_2(t))$ denote the solutions of (7), (11) and (12) initiating from $(x_0,y_0)$, respectively. Then $Y(t)\rightarrow 0$ as $t\rightarrow \infty$ and $Y_1(t)\le Y(t)\le Y_2(t)$ for all $t\ge 0$.

#### Proof

Let *p*(*t*), $p_1(t)$ and $p_2(t)$ denote the infection densities of systems (7), (11) and (12) at the initial state $(x_0,y_0)$, respectively. We first show that $p_1(t)\le p(t)\le p_2(t)$. By (5) we have

$$\begin{aligned} \frac{dp_1(t)}{dt}=p_1(t)(1-p_1(t))\left( b_I-\delta _I+\delta _U-{\bar{b}}_U(1-p_1)\right) , \\ \frac{dp_2(t)}{dt}=p_2(t)(1-p_2(t))\left( b_I-\delta _I+\delta _U-{\hat{b}}_U(1-p_2)\right) . \end{aligned}$$

Then both $p_1(t)$ and $p_2(t)$ increase in *t* from the initial infection density $x_0/(x_0+y_0)$. Since ${\bar{b}}_U< {\hat{b}}_U$, we find that $\frac{dp_1(t)}{dt}|_{(x_0,y_0)}>\frac{dp_2(t)}{dt}|_{(x_0,y_0)}$, and there exists $t_1>0$ such that $p_1(t)>p_2(t)$ for $0\le t\le t_1$. If $p_1(t)<p_2(t)$ for some $t>t_1$, we set $t_2=\inf \{t|p_1(t)<p_2(t)\}$. Then $p_1(t_2)=p_2(t_2)$ and $\frac{dp_1(t)}{dt}\le \frac{dp_2(t)}{dt}$ at the intersection. However, by the expressions of $\frac{dp_1}{dt}$ and $\frac{dp_2}{dt}$ we get that $\frac{dp_1(t)}{dt}>\frac{dp_2(t)}{dt}$ at any point in the first quadrant, which gives a contradiction. Thus $p_1(t)>p_2(t)$ for all $t>0$. By using the same idea to compare *p*(*t*) with $p_1(t)$ and $p_2(t)$, respectively, we can derive that $p_1(t)\ge p(t)\ge p_2(t)$ for $t>0$.

Now we prove $Y_1(t)\le Y_2(t)$ for $t\ge 0$. By (11) and (12) we see that $\frac{dY_1(t)}{dt}<\frac{dY_2(t)}{dt}$ at the initial state $(x_0,y_0)$. Then there exists $t_3$ such that $Y_1(t)<Y_2(t)$ for $0\le t\le t_3$. If $Y_1(t)>Y_2(t)$ for some $t>t_3$, we set $t_4=\inf \{t|Y_1(t)>Y_2(t)\}$. Then $Y_1(t_4)=Y_2(t_4)$ and $\frac{dY_1(t)}{dt}>\frac{dY_2(t)}{dt}$ at the intersection. It follows from $p_1(t_4)\ge p_2(t_4)$ and $Y_1(t_4)=Y_2(t_4)$ that $X_1(t_4)\ge X_2(t_4)$. Then by the expressions of $\frac{dY_1}{dt}$ and $\frac{dY_2}{dt}$ in (11) and (12), we derive $\frac{dY_1(t_4)}{dt}<\frac{dY_2(t_4)}{dt}$, which contradicts the assumption $Y_1(t)>Y_2(t)$ for some $t>t_3$. Thus $Y_1(t)<Y_2(t)$. By comparing *Y*(*t*) with $Y_1(t)$ and $Y_2(t)$, respectively, we obtain that $Y_1(t)\le Y(t)\le Y_2(t)$ for all $t\ge 0$. Since $Y_1(t)\rightarrow 0$ and $Y_2(t)\rightarrow 0$ as $t\rightarrow \infty$, we have $Y(t)\rightarrow 0$ as $t\rightarrow \infty$ $\square$

**Fig. 2:**
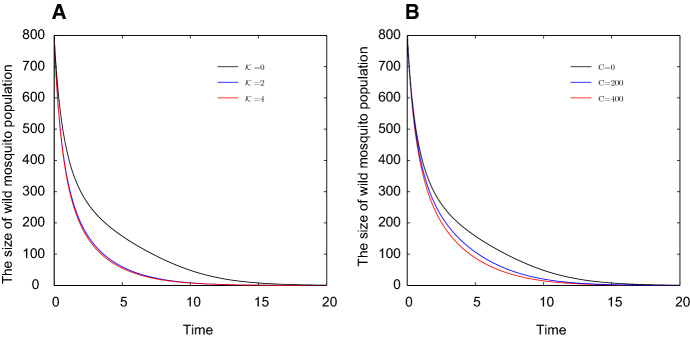
The declines of wild mosquito abundance under different release strategies. Let $b_I=0.45$, $b_U=0.55$, $\delta _I=0.05$, $\delta _U=0.048$, $d=0.001$. Set the initial state $(x_0,y_0)=(500,800)$. Panel a shows the number of wild mosquitoes changes with time *t* in different release ratios. Panel b shows the number of wild mosquitoes decreases with time *t* in different constant release amounts (Color figure online)

Figure 2a shows that if the release ratio $2<{\mathcal {K}}(t)<4$, then the curve for wild mosquito abundance lies in the very narrow area sandwiched by the red and blue curves. Another common release strategy is to release infected mosquitoes by a compensation policy such that the loss of infected mosquitoes is compensated by new releasing, then we assume $R(t)=C$ is a constant function for $t>0$ Yu (2018). In this case, we have

13 $$\begin{aligned} \left\{ \begin{array}{ll} \displaystyle \frac{\mathrm{d}x}{\mathrm{d}t}=(b_I-\delta _I)x-dx(x+y),\\ \displaystyle \frac{\mathrm{d}y}{\mathrm{d}t}=b_Uy\frac{y}{x+y+C}-\delta _Uy-dy(x+y). \end{array} \right. \end{aligned}$$

The discussions in Theorem 1 are applicable for system (13). Figure 2B shows the number of wild mosquitoes decreases with *t* under different constant release amount. If $200<C<400$, the curve of wild mosquito abundance lies in the narrow area sandwiched by the blue and red curves.

### Spray Pesticides

Here we consider the use of pesticides based on population replacement model (1). Then model (4) is reduced to

14 $$\begin{aligned} \left\{ \begin{array}{ll} \displaystyle \frac{\mathrm{d}x}{\mathrm{d}t}=(b_I-\delta _I-\phi _I(t))x-dx(x+y),\\ \displaystyle \frac{\mathrm{d}y}{\mathrm{d}t}=b_Uy\frac{y}{x+y}-(\delta _U+\phi _U(t))y-dy(x+y). \end{array} \right. \end{aligned}$$

It is natural to assume that $\phi _I(t)$ and $\phi _U(t)$ increase or decrease simultaneously. Define

15 $$\begin{aligned} a(t)=b_I-\delta _I-\phi _I(t)+\delta _U+\phi _U(t). \end{aligned}$$

Then the infection density can be expressed by

16 $$\begin{aligned} \frac{dp(t)}{dt}=p(t)(1-p(t))\left( a(t)-b_U(1-p)\right) . \end{aligned}$$

By (16), we obtain that *p*(*t*) increases in *t* if the initial infection density $p_0=\frac{x_0}{x_0+y_0}$ satisfies

17 $$\begin{aligned} \min \{a(t)\}>b_U(1-p_0), ~~\text {or equivalently,} ~~p_0>1-\frac{\min \{a(t)\}}{b_U}. \end{aligned}$$

Assume that $\phi _U(t)-\phi _I(t)$ takes the maximum value and the minimum value at time $t_1^*$ and $t_2^*$, respectively. Then

18 $$\begin{aligned} \max \{a(t)\}=a(t_1^*)~~~\text {and}~~~\min \{a(t)\}=a(t_2^*). \end{aligned}$$

Define

19 $$\begin{aligned} {\hat{\phi }}_U=\phi _U(t_1^*),~~{\bar{\phi }}_U=\phi _U(t_2^*),~~ {\hat{\phi }}_I=\phi _I(t_1^*)~~\text {and}~~{\bar{\phi }}_I=\phi _I(t_2^*). \end{aligned}$$

Construct the following systems

20 $$\begin{aligned}&\left\{ \begin{array}{ll} \displaystyle \frac{\mathrm{d}x}{\mathrm{d}t}=(b_I-\delta _I-{\bar{\phi }}_I)x-dx(x+y),\\ \displaystyle \frac{\mathrm{d}y}{\mathrm{d}t}=b_Uy\frac{y}{x+y}-(\delta _U+{\bar{\phi }}_U)y-dy(x+y). \end{array} \right. \end{aligned}$$

21 $$\begin{aligned}&\left\{ \begin{array}{ll} \displaystyle \frac{\mathrm{d}x}{\mathrm{d}t}=(b_I-\delta _I-{\hat{\phi }}_I)x-dx(x+y),\\ \displaystyle \frac{\mathrm{d}y}{\mathrm{d}t}=b_Uy\frac{y}{x+y}-(\delta _U+{\hat{\phi }}_U)y-dy(x+y). \end{array} \right. \end{aligned}$$

#### Remark 2.1

Suppose that (17) hold. Let *p*(*t*), $p_1(t)$ and $p_2(t)$ denote the infection densities of (7), (20) and (21) at the initial state $(x_0,y_0)$, respectively. As spraying pesticides only affects the term *a*(*t*) in (16), the density infection of system (7) with the measure of spraying pesticides satisfies $p_1(t)<p(t)< p_2(t)$ for all $t\ge 0$.

From (5), we see that *p*(*t*) is not affected by the use of insecticides if $\phi _I(t)\equiv \phi _U(t)$.

#### Remark 2.2

Suppose that (17) hold and $\phi _I(t)\equiv \phi _U(t)$. Redefine ${\bar{\phi }}_I=\min \{\phi _I(t)\}$, ${\bar{\phi }}_U=\min \{\phi _U(t)\}$ in (20) and ${\hat{\phi }}_I=\max \{\phi _I(t)\}$, ${\hat{\phi }}_U=\max \{\phi _U(t)\}$ in (21). Let (*X*(*t*), *Y*(*t*)), $(X_1(t),Y_1(t))$ and $(X_2(t),Y_2(t))$ denote the solutions of (7), (20) and (21) initiating from $(x_0,y_0)$, respectively. Then we have $Y(t)\rightarrow 0$ as $t\rightarrow \infty$ and $Y_1(t)\le Y(t)\le Y_2(t)$ for all $t\ge 0$ by the same method in Theorem 1.

When $\phi _I(t)=\phi _U(t)=\phi (t)$, we use an example to show the estimations of $\phi (t)$ and its effect on mosquito control. Assume that pesticides are sprayed every 7 days and the residual effects last for $T_1$ days with $3<T_1<4$. Assume that $\phi (t)$ is sandwiched by $y=y_1(t)$ and $y=y_2(t)$ with

$$\begin{aligned} y_1=\left\{ \begin{array}{ll} -\frac{0.3}{4}(t-4),~~nT<t<nT+4,\\ ~~~~0,~~~~nT+4<t<(n+1)T, \end{array} \right. ~~~y_2=\left\{ \begin{array}{ll} -\frac{0.2}{3}(t-3),~~nT<t<nT+3,\\ ~~~~0,~~~~nT+3<t<(n+1)T, \end{array} \right. \end{aligned}$$

**Fig. 3:**
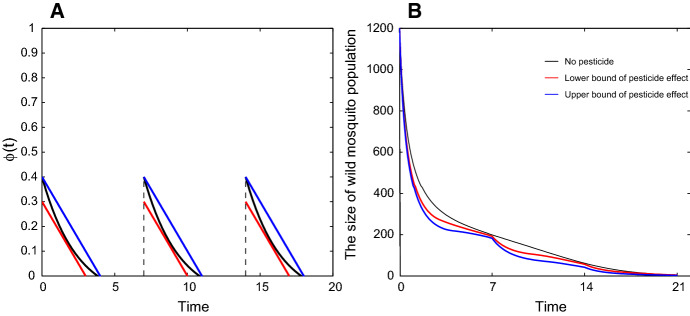
Estimations of $\phi (t)$ and its effect on mosquito control. **a** Let $T=7$. The black curve shows that $\phi (t)$ decreases with time after each spraying. The blue segments and red segments are the upper and lower bounds of $\phi (t)$, respectively. **b** Let $(x_0,y_0)=(500,1200)$ and the parameters be the same as in Fig. 2. The black curve shows the number of wild mosquitoes decreases without spraying pesticides, and the blue and red curves show the numbers of wild mosquitoes decrease with time when $\phi (t)=y_1(t)$ and $\phi (t)=y_2(t)$, respectively. The actual curve of wild mosquito abundance is sandwiched by the blue curve and red curve (Color figure online)

$n=0,1,2,\cdots$ (See Fig. 3A). Let $b_I=0.45$, $b_U=0.55$, $\delta _I=0.05$, $\delta _U=0.048$, $d=0.001$ and $T=7$. The actual curve of wild mosquito abundance under the measure of spraying pesticides is sandwiched by the blue and red curves in Fig. 3B, which represent the cases that $\phi (t)=y_1(t)$ and $\phi (t)=y_1(t)$, respectively.

#### Remark 2.3

**Fig. 4:**
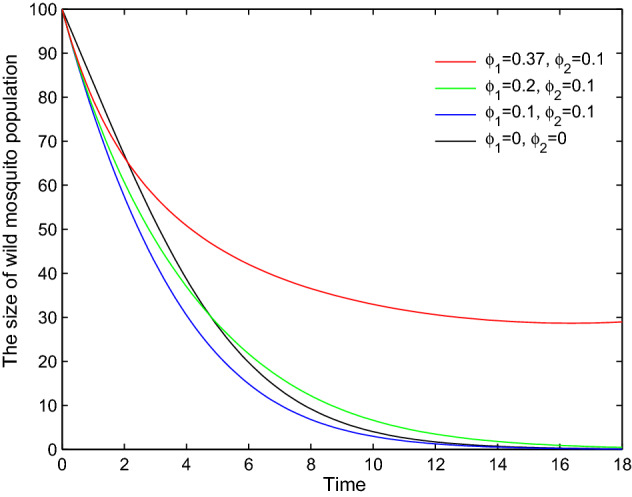
Pesticides may slow down the decline of the wild mosquito abundance. Let $b_I=0.45$, $b_U=0.55$, $\delta _I=0.05$, $\delta _U=0.048$, $d=0.001$ and $(x_0,y_0)=(200, 100)$. The black curve shows the number of wild mosquitoes changes with time *t* without spraying pesticides. If $\phi _I=\phi _U=0.1$, the blue curve shows wild mosquitoes decreases faster than the previous case. If we keep $\phi _U=0.1$ and increase $\phi _I$ to 0.2, the green curve shows the abundance of wild mosquitoes decreases faster than no-pesticide case at first, but it decreases slower after a while. If we continue to increase $\phi _I$ to 0.37 without changing $\phi _U$, the red curve shows the wild mosquitoes cannot be eradicated (Color figure online)

When $\phi _I(t) \not \equiv \phi _U(t)$, the relation among *Y*(*t*), $Y_1(t)$ and $Y_2(t)$ is uncertain. Although the use of pesticides increases the death rate of wild mosquitoes, it may slow down the decline of the wild mosquito population if the damage of insecticides to infected mosquitoes is greater than that to uninfected mosquitoes (See Fig. 4).

### Combine the Release of Infected Sterile Mosquitoes and Spraying Pesticides.

In this part, we discuss model (4). Recalling the definitions of *a*(*t*) and $b_U(t)$ in (10), (15) and (18), we have

22 $$\begin{aligned} \frac{dp(t)}{dt}=p(t)(1-p(t))\left( a(t)-b_U(t)(1-p)\right) , \end{aligned}$$

where ${\bar{a}}=a(t_1^*)\le a(t) \le a(t_2^*)={\hat{a}}$ and ${\bar{b}}_U\le b_U(t) \le \hat{b}_U$. Construct the following systems

23 $$\begin{aligned} \begin{aligned} \frac{d{\bar{p}}(t)}{dt}={\bar{p}}(t)(1-{\bar{p}}(t))\left( {\bar{a}}-{\hat{b}}_U(1-{\bar{p}}(t))\right) ,\\ \frac{d{\hat{p}}(t)}{dt}={\hat{p}}(t)(1-{\hat{p}}(t))\left( {\hat{a}}-{\bar{b}}_U(1-{\hat{p}}(t))\right) . \end{aligned} \end{aligned}$$

#### Remark 2.4

Let the initial state $(x_0,y_0)$ satisfies $p(0)=x_0/(x_0+y_0)>1-{\bar{a}}/{\hat{b}}$. By the similar comparison method in Theorem 1 we can obtain ${\bar{p}}(t)\le p(t) \le {\hat{p}}(t)$.

Now we consider the case that to release infected mosquitoes and to spray pesticides separately, and the release ratio and spraying density are fixed. Let $$t_0$$, $$t_1$$, $$t_2$$, $$\cdots $$ be the switch times between the two stages. Then the durations in all stages are $$[t_0, t_1)$$, $$[t_1, t_2)$$, $$[t_2, t_3)$$, $$\cdots $$. In view of the discussion in Remark 2.3, we assume $$\phi _I(t)<\phi _U(t)$$. Redefine $${\hat{b}}_U=b_U$$ and $${\hat{a}}=b_I-\delta _I-\phi _I(t)+\delta _U+\phi _U(t)$$ in spraying stage, and $${\bar{b}}_U= b_U/(1+{\mathcal {K}}(t))$$ and $${\bar{a}}=b_I-\delta _I+\delta _U$$ in releasing stage. Then $${\bar{a}}<{\hat{a}}$$ and $${\bar{b}}_U<{\hat{b}}_U$$. Construct systems

24 $$\begin{aligned} \frac{dp_1(t)}{dt}= & {} p_1(t)(1-p_1(t))\left( {\hat{a}}-{\hat{b}}_U(1-p_1(t))\right) , \end{aligned}$$

25 $$\begin{aligned} \frac{dp_2(t)}{dt}= & {} p_2(t)(1-p_2(t))\left( {\bar{a}}-{\bar{b}}_U(1-p_2(t))\right) . \end{aligned}$$

Let *p*(*t*) denote the *Wolbachia* infection density of systems (22). Define

26 $$\begin{aligned} \beta _1=1-{\hat{a}}/{\hat{b}}_U, \quad \beta _2=1-{\bar{a}}/{\bar{b}}_U, \quad p_1^*=\min \{\beta _1, \beta _2\}, \quad p_2^*=\max \{\beta _1, \beta _2\}. \end{aligned}$$

Then we have the following estimations for *p*(*t*).

#### Theorem 2

Suppose that $p(0)>p_2^*$, ${\bar{b}}_U<{\hat{b}}_U$ and ${\bar{a}}<{\hat{a}}$. Let system (22) switches between systems (24) and (25) and the staying times in the two systems are $T_1$ and $T_2$, respectively. If $T_1$ and $T_2$ satisfy $0<{\bar{T}}_1<T_1<{\hat{T}}_1<\infty$ and $0<{\bar{T}}_2<T_2<{\hat{T}}_2<\infty$, then we have the following result:

1. If $p(0)\ge \beta _3 =1-({\hat{a}}-{\bar{a}})/({\hat{b}}_U-{\bar{b}}_U)$, then $p_2(t)\le p(t)\le p_1(t)$ for all $t>0$.
2. If $0<p(0)<\beta _3$, then there exists a $t^*>0$ such that $p_1(t)< p_2(t)$ when $0<t<t^*$, and $p_1(t)> p_2(t)$ when $t> t^*$. Denote by $\mathcal {D}_1$ the area enclosed by $p_1(t)$ and $p_2(t)$ for $t> t^{*}$. Then $p=p(t)$ enters $\mathcal {D}_1$ for sufficiently large *t* and stays in this area thereafter.

#### Proof

(1) Since $p_1(0)=p_2(0)=p(0)>p_2^*$, all the three functions *p*, $p_1$, and $p_2$ increase in $t>0$. Define

27 $$\begin{aligned} g_0(p)={\hat{a}}-{\hat{b}}_U+{\hat{b}}_Up-({\bar{a}}-{\bar{b}}_U+{\bar{b}}_Up). \end{aligned}$$

It can be easily verified that

28 $$\begin{aligned} p>\beta _3 \qquad \mathrm{if \,\,\, and\,\,\, only\,\,\, if\qquad } g_0(p)>0. \end{aligned}$$

Hence at $t=0$, it holds that

29 $$\begin{aligned}&p_1'(0)-p_2'(0)=p(0)(1-p(0))g_0(p(0))>0. \end{aligned}$$

Therefore, $p_1(t)>p_2(t)$ for small $t>0$. Indeed, this relation remains valid for all $t>0$; otherwise, there would be some $\tau >0$ such that $p_1(t)>p_2(t)$ for $0<t<\tau$, but $p_1(\tau )=p_2(\tau )$. Hence $p_1'(\tau )\le p_2'(\tau )$, which contradicts the fact that (29) is still valid if *p*(0) is replaced with $p_1(\tau )$. It follows that $p_1(t)>p_2(t)$ for all $t>0$. Define

30 $$\begin{aligned} r(t)=\ln \frac{p(t)}{1-p(t)}. \end{aligned}$$

To confirm the relation of *p*(*t*) with $p_1(t)$ and $p_2(t)$, we denote by $r_1(t)$ and $r_2(t)$ the corresponding forms of *r*(*t*) defined in (30) where *p* is replaced by $p_1$ or $p_2$. Due to the monotonic dependance of *r*(*t*) on *p*(*t*), $p(t)\le p_1(t)$ is equivalent to $r(t)\le r_1(t)$. Suppose for contradiction that $r(t)>r_1(t)$ at some *t*. Then

31 $$\begin{aligned} {\hat{t}}=\inf \{t|r(t)>r_1(t)\} \end{aligned}$$

is finite. We claim that *p*(*t*) is governed by system (25) at time ${\hat{t}}$. If this is not true, then there exists an $\epsilon >0$ such that the system stays in System (24) for $t\in [{\hat{t}},{\hat{t}}+\epsilon )$, and therefore, $r(t)=r_1(t)$ in this interval, which contradicts the definition of ${\hat{t}}$. We now show that the system does not stay in System (25) at time ${\hat{t}}$ either. If it does, then by (31) and $r_1(0)=r(0)$ we find that $r_1({\hat{t}})=r({\hat{t}})$ and so the right-hand derivative of *r*(*t*) must not be less than that of $r_1(t)$ at ${\hat{t}}$, i.e., $r_{+}'({\hat{t}})\ge r_{1+}'({\hat{t}})$. In addition, by taking derivatives of $r_1(t)$ and $r_2(t)$, we find

$$\begin{aligned} r_1'(t)={\hat{a}}-{\hat{b}}_U+{\hat{b}}_Up_1(t)\quad \mathrm{and}\quad r_2'(t)={\bar{a}}-{\bar{b}}_U+{\bar{b}}_Up_2(t). \end{aligned}$$

It then follows that

$$\begin{aligned} r_{1+}'({\hat{t}})- r_{+}'({\hat{t}}) ={\hat{a}}-{\hat{b}}_U+{\hat{b}}_Up_1({\hat{t}})-({\bar{a}}-{\bar{b}}_U+{\bar{b}}_Up_1({\hat{t}})) =g_0({\hat{t}})>0, \end{aligned}$$

which gives a contradiction. Thus

$$\begin{aligned} r(t)\le r_1(t) \quad \mathrm{and} \quad p(t)\le p_1(t) \end{aligned}$$

for all $t>0$. The same reasoning shows that $p(t)\ge p_2(t)$.

(2) Define

32 $$\begin{aligned} s(t)=r_1(t)-r_2(t). \end{aligned}$$

Clearly, $s(0)=0$. Since $p(0)<\beta _3$, we have

$$\begin{aligned} s'(0)={\hat{a}}-{\hat{b}}_U+{\hat{b}}_Up(0)-({\bar{a}}-{\bar{b}}_U+{\bar{b}}_Up(0))=g_0(0)<0. \end{aligned}$$

Hence $s(t)<0$, or equivalently, $p_1(t)<p_2(t)$, for all small $t>0$. In addition, since both $p_1(t)$ and $p_2(t)$ approach 1 as $t\rightarrow \infty$, we have

$$\begin{aligned} \lim _{t\rightarrow \infty } s'(t)=\lim _{t\rightarrow \infty }\left( {\hat{a}}-{\hat{b}}_U+{\hat{b}}_Up_1(t)-({\bar{a}}-{\bar{b}}_U+{\bar{b}}_Up_2(t))\right) = {\hat{a}}-{\bar{a}}>0. \end{aligned}$$

Thus $s(t)\rightarrow \infty$ as $t\rightarrow \infty$, and $p_1(t)>p_2(t)$ for all *t* sufficiently large. It follows that $p_1(t)$ and $p_2(t)$ must coincide at some $t>0$. Let $t^*>0$ be the least time at which $p_1(t)=p_2(t)$, or equivalently, $s(t)=0$. Then

$$\begin{aligned} s'(t^*)={\hat{a}}-{\hat{b}}_U+{\hat{b}}_Up_1(t^*)-({\bar{a}}-{\bar{b}}_U+{\bar{b}}_Up_1(t^*))=g_0(p_1(t^*))\ge 0. \end{aligned}$$

By (28), we see that $$p_1(t^*)=p_2(t^*)\ge \beta _3>p_2^*$$. As both $$p_1$$ and $$p_2$$ increase for $$t>0$$, we have $$p_1(t)>\beta _3$$ and $$p_2(t)>\beta _3$$ for $$t>t^*$$. Hence $$p_1$$ and $$p_2$$ cannot meet at another time after $$t=t^*$$ since at any possible intersection point it follows from (28) that $$ s'>0$$. Note that $$p_1(t^*)=p_2(t^*)\ge \max \{\beta _1,\beta _2,\beta _3\}$$. By using the same argument in the proof of Part (1), we can show that if $$p_2(t_1)\le p(t_1)\le p_1(t_1)$$ at any $$t_1>t^*$$, then this ordering will be maintained for all $$t>t_1$$. In other words, if $$p=p(t)$$ enters the area $$\mathcal {D}_1$$ at any $$t>t^*$$, then it will stay in this area thereafter. We now show that $$p=p(t)$$ enters the area $$\mathcal {D}_1$$ when *t* is sufficiently large. Without loss of generality, we assume that

$$\begin{aligned} p(t^*)<p_1(t^*)=p_2(t^*). \end{aligned}$$

Define

33 $$\begin{aligned} s_1(t)=r(t)-r_2(t). \end{aligned}$$

Then $s_1(t^*)<0$. It remains to show that $s_1$ becomes positive at some $t>t^*$. Due to the monotonicity of $g_0(p)$ in *p*, and $g_0(p)=0$ when $p=\beta _3$, there is an $\varepsilon >0$ such that $g_0(p)>\varepsilon$ when $p>(1+\beta _3)/2$. Let

$$\begin{aligned} \beta _4=\max \left\{ \frac{1+\beta _3}{2},\, 1-\frac{\varepsilon }{2{\bar{b}}_U}, \, 1-\frac{\varepsilon {\bar{T}}_1}{4{\hat{T}}_2 {\bar{b}}_U}\right\} . \end{aligned}$$

Then $\beta _3<\beta _4<1$. Since *p*(*t*) increases and approaches 1 as $t\rightarrow \infty$, there is a unique $t_4>0$ such that $\min \{p(t_4),p_1(t_4),p_2(t_4)\}=\beta _4$.

If the system stays in System (24) at $t>t_4$, then

$$\begin{aligned} p_2(t)-p(t)<1-\left( 1-\frac{\varepsilon }{2{\bar{b}}_U}\right) =\frac{\varepsilon }{2{\bar{b}}_U}, \end{aligned}$$

and therefore

34 $$\begin{aligned} s'_1(t)= & {} {\hat{a}}-{\hat{b}}_U+{\hat{b}}_Up-({\bar{a}}-{\bar{b}}_U+{\bar{b}}_Up_2)\nonumber \\> & {} {\hat{a}}-{\hat{b}}_U+{\hat{b}}_Up-\left( {\bar{a}}-{\bar{b}}_U+{\bar{b}}_U\left( p+\frac{\varepsilon }{2{\bar{b}}_U}\right) \right) \nonumber \\= & {} g_0(p)-\frac{\varepsilon }{2}>\frac{\varepsilon }{2}. \end{aligned}$$

If the system stays in System (25) at $t>t_4$, then

35 $$\begin{aligned} s'_1(t)={\bar{a}}-{\bar{b}}_U+{\bar{b}}_Up-({\bar{a}}-{\bar{b}}_U+{\bar{b}}_Up_2) >-{\bar{b}}_U\frac{\varepsilon {\bar{T}}_1}{4{\hat{T}}_2 {\bar{b}}_U}=-\frac{\varepsilon {\bar{T}}_1}{4{\hat{T}}_2 }. \end{aligned}$$

Suppose that $$[t_0^*,t_0^*+T_1)$$ is a spraying stage and $$[t_0^*+T_1,t_0^*+T_1+T_2)$$ is a releasing stage with $$t_0^*>t_4$$. Note that

$$\begin{aligned} s_1(t_0^*+T_1+T_2)= & {} s_1(t_0^*+T_1+T_2)-s_1(t_0^*)+s_1(t_0^*)\\\ge & {} \frac{\varepsilon }{2}T_1-\frac{\varepsilon {\bar{T}}_1}{4{\hat{T}}_2}T_2+s_1(t_0^*)\\\ge & {} \frac{\varepsilon }{4}T_1+s_1(t_0^*). \end{aligned}$$

It is then clear that $s_1(t)\rightarrow \infty$ as $t\rightarrow \infty$, and so $s_1(t)$ becomes positive for large *t*. The proof is completed. $\square$

## Mosquito Control Under the Effect of Heatwave

In high-temperature condition, mosquitoes may lose *Wolbachia* according to (Ross et al. 2020, 2017). Let $\mu$ ($0<\mu <1$) denote the imperfect transmission rate. By making minor changes in (1), we obtain the system

36 $$\begin{aligned} \left\{ \begin{array}{ll} \displaystyle \frac{\mathrm{d}x}{\mathrm{d}t}=b_I(1-\mu )x-\delta _Ix-dx(x+y),\\ \displaystyle \frac{\mathrm{d}y}{\mathrm{d}t}=b_I\mu x+b_Uy\frac{y}{x+y}-\delta _Uy-dy(x+y). \end{array} \right. \end{aligned}$$

If $b_I(1-\mu )-\delta _I-d\le 0$, the infected mosquitoes will die out naturally and the mosquito population replacement is going to fail. So we assume

37 $$\begin{aligned} b_I(1-\mu )-\delta _I-d>0 \end{aligned}$$

in the following discussion. Clearly, $E_1=(0,(b_U-\delta _U)/d)$ is an infection-free equilibrium and its local stability is determined by the Jacobian of (36),

38 $$\begin{aligned} DF(x,y)= \begin{gathered} \begin{pmatrix} b_I(1-\mu )-\delta _I-2dx-dy &{} -dx \\ b_I\mu -\frac{b_Uy^2}{(x+y)^2}-dy &{} \frac{b_Uy(2x+y)}{(x+y)^2}-\delta _U-dx-2dy \end{pmatrix}. \end{gathered} \end{aligned}$$

At the infection-free equilibrium point, we have

39 $$\begin{aligned} DF\left( 0,\frac{b_U-\delta _U}{d}\right) = \begin{gathered} \begin{pmatrix} b_I(1-\mu )-\delta _I-b_U+\delta _U &{} 0 \\ b_I\mu -2b_U+\delta _U &{} \delta _U-b_U \end{pmatrix}. \end{gathered} \end{aligned}$$

This matrix has the eigenvalues $(b_I-b_U)+(\delta _U-\delta _I)-b_I\mu$ and $\delta _U-b_U$. From (2) and (3), we see that both eigenvalues are negative, and so $E_1$ is locally asymptotically stable. To obtain the positive equilibrium in the first quadrant, we solve equations

$$\begin{aligned} \left\{ \begin{array}{ll} \displaystyle b_I(1-\mu )x-\delta _Ix-dx(x+y)=0,\\ \displaystyle b_I\mu x+b_Uy\frac{y}{x+y}-\delta _Uy-dy(x+y)=0. \end{array} \right. \end{aligned}$$

The first equation gives

40 $$\begin{aligned} x+y=(b_I(1-\mu )-\delta _I)/d:=\kappa _1. \end{aligned}$$

Substituting (40) into the second equation yields

41 $$\begin{aligned} Ay^2-By+C=0, \end{aligned}$$

where

42 $$\begin{aligned} A=b_U/\kappa _1,~~~~~ B=\delta _U+d\kappa _1+b_I\mu ~~~~~\text {and}~~~~~C=b_I\mu \kappa _1. \end{aligned}$$

The discriminant of (41)

$$\begin{aligned} \Delta= & {} B^2-4AC=(b_I\mu +d\kappa _1+\delta _U)^2-4b_Ib_U\mu \\ {}= & {} (b_I\mu +b_I(1-\mu )-\delta _I+\delta _U)^2-4b_Ib_U\mu =(b_I-\delta _I+\delta _U)^2-4b_Ib_U\mu . \end{aligned}$$

Define

43 $$\begin{aligned} \mu ^*=(b_I-\delta _I+\delta _U)^2/4b_Ib_U. \end{aligned}$$

It follows from (2) and (3) that $(b_I-\delta _I+\delta _U)^2<4b_Ib_U$, implying $0<\mu ^*<1$. If we regard $\Delta$ as a function of $\mu$, then $\Delta (\mu )$ decreases in $\mu$ and $\Delta (\mu ^*)=0$. When $\mu >\mu ^*$, there is no positive equilibrium in the first quadrant and $E_1$ is the only stable equilibrium. In this case *Wolbachia* frequency declines to zero. When $\mu <\mu ^*$, (41) has the solutions

44 $$\begin{aligned} y_1=\frac{B-\sqrt{\Delta }}{2A}~~~~~\text {and}~~~~~y_2=\frac{B+\sqrt{\Delta }}{2A}, \end{aligned}$$

and both the solutions are nonnegative since $\Delta <B^2$. By (40), we obtain the two equilibria

45 $$\begin{aligned} (x_1,y_1)=\left( \kappa _1-\frac{B-\sqrt{\Delta }}{2A},\frac{B-\sqrt{\Delta }}{2A}\right) ,~~ (x_2,y_2)=\left( \kappa _1-\frac{B+\sqrt{\Delta }}{2A},\frac{B+\sqrt{\Delta }}{2A}\right) .\nonumber \\ \end{aligned}$$

**Fig. 5:**
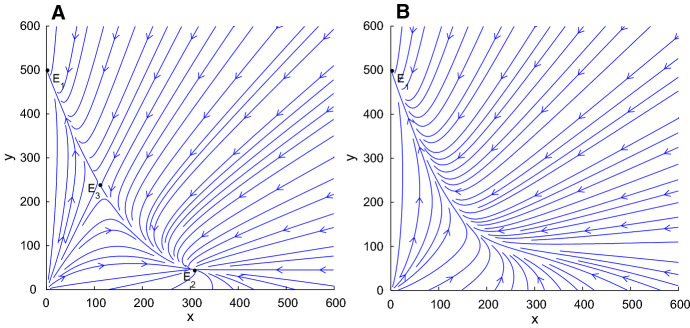
The vector field direction of system (36). Let $b_I=0.45$, $\mu =0.11$, $b_U=0.55$, $\delta _I=0.05$, $\delta _U=0.048$, $d=0.001$. Panel a shows that system (36) has equilibria $E_1$, $E_2$ and $E_3$. Compared with system (1), $E_2$ becomes an interior equilibrium. Let $\mu$ increase to 0.25 without changing the other parameters. Panel b shows that system (36) only admits an equilibria $E_1$

It follows from

46 $$\begin{aligned} y_2= & {} \frac{b_I-\delta _I+\delta _U+\sqrt{(b_I-\delta _I+\delta _U)^2-4b_Ib_U\mu }}{2\frac{b_U}{\kappa _1}}\nonumber \\= & {} \frac{b_I(1-\mu )-\delta _I}{2b_Ud}\left( b_I-\delta _I+\delta _U+ \sqrt{(b_I-\delta _I+\delta _U)^2-4b_Ib_U\mu }\right) \end{aligned}$$

that $y_2$ decreases in $\mu$. Then $E_2(x_1,y_1)$ and $E_3(x_2,y_2)$ stay in the first quadrant when $\mu <\mu ^*$. As in Farkas and Hinow (2010), we give numerical examples to show the vector field (See Fig. 5).

To offset the negative effect of heatwave on mosquito control, we employ the measures of releasing infected sterile mosquitoes and spraying pesticides to control wild mosquitoes as in the previous Section. By taking into account the first measure, we have

47 $$\begin{aligned} \left\{ \begin{array}{ll} \displaystyle \frac{\mathrm{d}x}{\mathrm{d}t}=b_I(1-\mu )x-\delta _Ix-dx(x+y),\\ \displaystyle \frac{\mathrm{d}y}{\mathrm{d}t}=b_I\mu x+b_Uy\frac{y}{x+y+R(t)}-\delta _Uy-dy(x+y). \end{array} \right. \end{aligned}$$

### Theorem 3

Assume that (37) holds and $R(t)\equiv R$. Then the size of wild mosquito population can be suppressed to a level below ${\mathcal {S}}$ from any positive initial states if the release abundance satisfies the following three conditions:

(*i*) $$R>\frac{4Cb_U}{B^2}-\kappa _1$$;

(*ii*) $$R>\min \{\frac{b_U\kappa _1^2}{\kappa _1B-C}-\kappa _1,~ \frac{2b_U\kappa _1}{B}-\kappa _1\}$$;

(*iii*) $$R>\frac{b_U{\mathcal {S}}^2}{B{\mathcal {S}}-C}-\kappa _1$$ or $$R<\frac{2Sb_U}{B}-\kappa _1$$,

where $\kappa _1=(b_I(1-\mu )-\delta _I)/d$, *B* and *C* are defined in (42).

### Proof

Similar to the discussion of system (36), the interior equilibrium (*x*, *y*) of system (47) is the solution to equations $x+y=\kappa _1$ and

48 $$\begin{aligned} A_Ry^2-By+C=0, \end{aligned}$$

where $A_R=\frac{b_U}{\kappa _1+R}$, $B=\delta _U+d\kappa _1+b_I\mu$ and $C=b_I\mu \kappa _1$. The discriminant of (48) is

49 $$\begin{aligned} \Delta _R=B^2-4A_RC=(\delta _U+d\kappa _1+b_I\mu )^2-4\frac{b_U}{\kappa _1+R}\,b_I\mu \kappa _1. \end{aligned}$$

It is easy to verify that $\Delta _R$ increases in *R*, and $\Delta _R>0$ when condition (*i*) holds. In this case, (48) has two solutions

$$\begin{aligned} y_1=\frac{B-\sqrt{\Delta _R}}{2A_R}~~~~~\text {and}~~~~~y_2=\frac{B+\sqrt{\Delta _R}}{2A_R}, \end{aligned}$$

and both the solutions are nonnegative. From the facts

$$\begin{aligned} y_1= & {} \frac{B-\sqrt{B^2-4A_RC}}{2A_R}=\frac{2C}{B+\sqrt{B^2-4A_RC}}\\= & {} \frac{2b_I\mu \kappa _1}{\delta _U+d\kappa _1+b_I\mu + \sqrt{(\delta _U+d\kappa _1+b_I\mu )^2-4\frac{b_U}{\kappa _1+R}b_I\mu \kappa _1}} \end{aligned}$$

and

$$\begin{aligned} y_2=\frac{B+\sqrt{B^2-4A_RC}}{2A_R}=\frac{(\delta _U+d\kappa _1+b_I\mu )+\sqrt{(\delta _U+d\kappa _1+b_I\mu )^2-4\frac{b_U}{\kappa _1+R}b_I\mu \kappa _1}}{\frac{2b_U}{\kappa _1+R}} \end{aligned}$$

we see that $y_1\rightarrow \frac{C}{B}=\frac{b_I\mu \kappa _1}{\delta _U+d\kappa _1+b_I\mu }<\kappa _1$ and $y_2\rightarrow \infty$ when $R\rightarrow \infty$. Then $(x_1,y_1)$ stays in the first quadrant and $(x_2,y_2)$ stays in the second quadrant when $y_2>\kappa _1$, which is equivalent to condition (*ii*). In this case $(x_1,y_1)$ is globally stable and the wild mosquitoes can be suppressed to a level below ${\mathcal {S}}$ if $y_1<{\mathcal {S}}$, which is equivalent to condition (*iii*). $\square$

Now we consider the measure of spraying pesticides and build the system

50 $$\begin{aligned} \left\{ \begin{array}{ll} \displaystyle \frac{\mathrm{d}x}{\mathrm{d}t}=b_I(1-\mu )x-(\delta _I+\phi _I) x-dx(x+y),\\ \displaystyle \frac{\mathrm{d}y}{\mathrm{d}t}=b_I\mu x+b_Uy\frac{y}{x+y}-(\delta _U+\phi _U)y-dy(x+y). \end{array} \right. \end{aligned}$$

Upon rescaling $\delta _I=\delta _I+\phi _I$ and $\delta _U=\delta _U+\phi _U$, this model is the same as system (36). Here we only discuss the case $\phi _I=\phi _U$ and the discussion for $\phi _I\ne \phi _U$ is similar. $\mu ^*$ defined in (43) is applicable here as $\phi _I-\phi _U=0$. If $b_I(1-\mu )-\delta _I-\phi _I-d<0$ or $\mu > \mu ^*$, the infected mosquitoes will die out. Spraying pesticides may suppress wild mosquitoes to a low level, but it takes little use of infected mosquitoes and requires a great deal of pesticides. We next consider the condition

51 $$\begin{aligned} \mu < \mu ^*~~~~~\text {and}~~~~~ b_I(1-\mu )-\delta _I-\phi -d>0 \end{aligned}$$

Define

$$\begin{aligned} A=\frac{db_U}{b_I(1-\mu )-\delta _I-\phi },~~~B=\delta _U+b_I-\delta _I, ~~~C=\frac{b_I\mu }{d}(b_I(1-\mu )-\delta _I-\phi ). \end{aligned}$$

### Theorem 4

Let $\phi$ be the excess death rate of both infected and uninfected mosquitoes caused by pesticides. Suppose that (51) holds. Then the size of wild mosquito population can be suppressed to a level below ${\mathcal {S}}$ if $y(0)/x(0)<y_2/x_2$ and

$$\begin{aligned}\phi >b_I(1-\mu )-\delta _I-\max \left\{ \frac{2db_U{\mathcal {S}}}{B-\sqrt{B^2-4b_Ib_U\mu }},~ \frac{2d{\mathcal {S}}b_U}{B}\right\} \end{aligned}$$

where

$$\begin{aligned} x_2=\frac{b_I(1-\mu )-\delta _I-\phi }{d}-\frac{B+\sqrt{B^2-4AC}}{2A},~~~y_2=\frac{B+\sqrt{B^2-4AC}}{2A}, \end{aligned}$$

### Proof

When $\mu < \mu ^*$, system (50) admits two interior equilibria $E_2(x_1,y_1)$ and $E_3(x_2,y_2)$ given by (45) if $\delta _I$ and $\delta _U$ are replaced by $\delta _I+\phi$ and $\delta _U+\phi$, respectively. As shown in Fig. 5A, $E_2(x_1,y_1)$ is locally stable while $E_3(x_2,y_2)$ is an unstable saddle point. Then the wild mosquitoes can be suppressed to the below ${\mathcal {S}}$ if the following two conditions hold: (*i*) $E_2$ stays below $y={\mathcal {S}}$; (*ii*) the initial state lies in the basin of attraction of $E_2$. It follows from (44) that condition (*i*) is equivalent to $\frac{B-\sqrt{B^2-4AC}}{2A}<{\mathcal {S}}$, which implies

$$\begin{aligned} \phi> & {} \min \left\{ b_I(1-\mu )-\delta _I-\frac{2db_U{\mathcal {S}}}{B-\sqrt{B^2-4b_Ib_U\mu }},~ b_I(1-\mu )-\delta _I-\frac{2d{\mathcal {S}}b_U}{B}\right\} \\= & {} b_I(1-\mu )-\delta _I-\max \left\{ \frac{2db_U{\mathcal {S}}}{B-\sqrt{B^2-4b_Ib_U\mu }},~ \frac{2d{\mathcal {S}}b_U}{B}\right\} \end{aligned}$$

Let *p*(*t*) denote the infection density in system (50). Then

52 $$\begin{aligned} \frac{\mathrm{d}p(t)}{\mathrm{d}t}= & {} \frac{x'y-xy'}{(x+y)^{2}} \nonumber \\= & {} \frac{xy}{(x+y)^{2}}\bigg (b_I(1-\mu )-\delta _I-b_I\mu \frac{x}{y}-b_U\frac{y}{x+y}+\delta _U\bigg ) \nonumber \\= & {} \frac{xy}{(x+y)^{2}}\bigg ((b_I(1-\mu )-\delta _I+\delta _U) -(b_I\mu \frac{x}{y}+b_U\frac{1}{x/y+1})\bigg ) \end{aligned}$$

Define

$$\begin{aligned} f(r)=b_I\mu r+\frac{b_U}{r+1}. \end{aligned}$$

Since $f'(r)=b_I\mu -b_U/(1+r)^2$, we see that $f'(r)$ increases in *r* when $r>0$ and $f'(r^*)=0$ with $r^*=\sqrt{\frac{b_U}{b_I\mu }}-1$. Then *f*(*r*) decreases in *r* when $0\le r\le r^*$ and increases in *r* when $r\ge r^*$. In addition, $f(0)=b_U$ and $f(r)\rightarrow \infty$ when $r\rightarrow \infty$. On the other hand, $E_2(x_1,y_1)$ and $E_3(x_2,y_2)$ are two interior equilibria, so $r_1=x_1/y_1$ and $r_2=x_2/y_2$ are two roots to equation

$$\begin{aligned} (b_I(1-\mu )-\delta _I+\delta _U)-f(r)=0. \end{aligned}$$

It follows from (52) that *p*(*t*) decreases in *t* if $x(t)/y(t)<x_2/y_2$ or $x(t)/y(t)>x_1/y_1$, and increases in *t* if $x_2/y_2<x(t)/y(t)<x_1/y_1$. Thus $y=\frac{y_2}{x_2}x$ is the separatrix of the basins of attraction of $E_2$ and $E_3$, implying that condition (*ii*) holds if the initial state (*x*(0), *y*(0)) satisfies $y(0)/x(0)<y_2/x_2$. $\square$

Finally, we consider applying the above two measures together to suppress wild mosquitoes under the effect of heatwave. The integrated model is given by

53 $$\begin{aligned} \left\{ \begin{array}{ll} \displaystyle \frac{\mathrm{d}x}{\mathrm{d}t}=b_I(1-\mu )x-(\delta _I+\phi ) x-dx(x+y),\\ \displaystyle \frac{\mathrm{d}y}{\mathrm{d}t}=b_I\mu x+b_Uy\frac{y}{x+y+R(t)}-(\delta _U+\phi )y-dy(x+y). \end{array} \right. \end{aligned}$$

By combining Theorem 3 and 4, we obtain the following result.

### Remark 3.1

Let $\phi$ be the excess death rate of both infected and uninfected mosquitoes caused by pesticides and $R(t)\equiv R$. Suppose that (51) holds. Then the size of wild mosquito population can be suppressed to a level below the safe threshold ${\mathcal {S}}$ if the release abundance and pesticide effect satisfy

$$\begin{aligned} (i)~B^2-4A_RC>0,~~~~ (ii)~\frac{B+\sqrt{B^2-4A_RC}}{2A_R}>\kappa _1~~~\text {and}~~~ (iii)~\frac{B-\sqrt{B^2-4A_RC}}{2A_R}<{\mathcal {S}}, \end{aligned}$$

$$\begin{aligned} \text {where}~~ \kappa _1=\frac{b_I(1-\mu )-\delta _I-\phi }{d},~~~A_R=\frac{b_U}{\kappa _1+R},~~~ B=\delta _U+b_I-\delta _I~~~ and ~~~C=b_I\mu \kappa _1. \end{aligned}$$

The proof follows from Theorem 3 and 4 directly.

## Discussion

Some mosquito-borne diseases, such as dengue fever, occur periodically and are triggered by imported patients in some sub-tropical areas. It is essential to take emergency measures when the dengue cases are large. As there is no vaccine or effective medication available, eliminating the transmission vector has been the most effective method. The traditional measure uses pesticides, which kills mosquitoes quickly but cannot suppress mosquitoes for a long time due to insecticide resistance. One promising complementary method is to use incompatible insect technique (IIT) by releasing *Wolbachia*-infected mosquitoes into wild mosquito populations, which has been proven to be a novel and environmental-friendly way for mosquito control. Many interesting models of difference or differential equations have been developed to investigate the dynamic behavior of wild mosquito populations based on IIT, see (Hu et al. 2019; Huang et al. 2018; Keeling et al. 2003; Shi and Yu 2020; Yu and Zheng 2019; Zhang et al. 2020; Zheng et al. 2014) and the references therein. The radiation-based sterile insect technique (SIT) uses radiation to sterilize male mosquitoes, leading irradiated males that are unable to produce offspring after mating with wild females. Mathematical models for studying the suppression effects on SIT have provided applicable guidance (Cai et al. 2014; Li and Yuan 2015; Yu 2020; Yu and Li 2019, 2020). Several interesting mathematical models have been developed to deal with mosquito control via a combination of *Wolbachia* and insecticides (Li and Liu 2020; Qu et al. 2018; Zheng et al. 2018). In this work, we introduced population suppression measures, releasing infected sterile mosquitoes and spraying pesticides, into population replacement model to discuss the mosquito control.

**Fig. 6:**
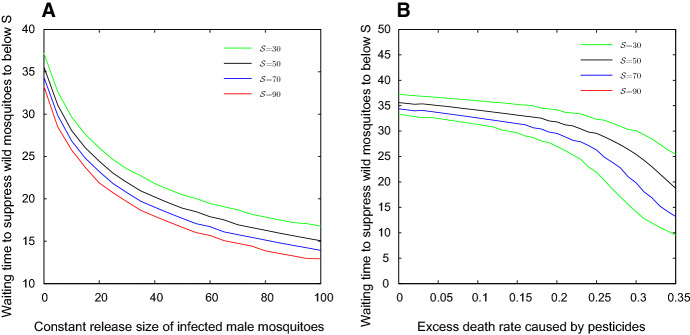
The waiting times to suppress wild mosquito abundance to a level below ${\mathcal {S}}$. Let $b_I=0.45$, $b_U=0.55$, $\delta _I=0.05$, $\delta _U=0.048$, $d=0.001$ and $(x_0,y_0)=(200,800)$. Panel a shows the waiting times to suppress wild mosquito abundance to a level below ${\mathcal {S}}$ under different releasing amounts, and panel b shows the waiting times under different pesticide effects (Color figure online)

In Sect. 2, we provided the estimations of wild mosquito abundance or infection density in normal environment under different control measures. Mosquito control is always affected by environmental conditions. In Hu et al. (2019), we considered the case that female mosquitoes lay diapause eggs in cold season in a sub-tropical area. In this work, we consider the opposite extreme case that infected mosquitoes may lose *Wolbachia* in a high-temperature season (Ross et al. 2020, 2017). Under the effect of heatwave, the offspring of infected females lose *Wolbachia* infection with positive leakage rate $\mu$. We discussed how to offset the effect of the heatwave in sect. 3. For example, we consider the case where $b_I=0.45$, $b_U=0.55$, $\delta _I=0.05$, $\delta _U=0.048$, $d=0.001$ and the initial state $(x_0,y_0)=(200,800)$. The wild mosquitoes can be replaced by infected mosquitoes totally without control measures in model (1). However, by releasing infected mosquitoes or spraying pesticides we can greatly increase the replacement speed. Figure 6 shows the waiting times to suppress wild mosquito abundance to a level below ${\mathcal {S}}$ with the measure of release infected mosquitoes (Figure 6A) or spraying pesticides (Figure 6B).

**Fig. 7:**
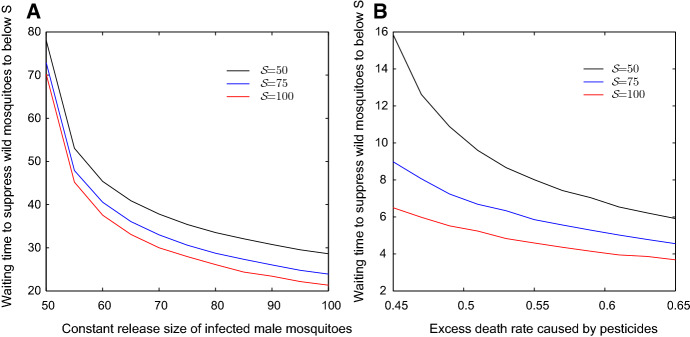
The waiting times to suppress wild mosquito abundance to a level below ${\mathcal {S}}$. Let the parameters and initial state be the same as in Fig. 6 and set $\mu =0.1$. Panel a shows the waiting times to suppress wild mosquito abundance to a level below ${\mathcal {S}}$ under different releasing amounts, and panel b shows the waiting times under different pesticide effects (Color figure online)

Let the parameters be the same as in Fig. 6 and set $\mu =0.1$. Then infected mosquitoes will die out in competing with wild uninfected mosquitoes in model (36). In this case, the extra measures must be taken to ensure the successful suppression of wild mosquitoes. Figure 7 shows the waiting time to suppress wild mosquito abundance to a level below ${\mathcal {S}}$ under the measures of releasing infected mosquitoes (Fig. 7A) or spraying pesticides (Fig. 7B). With the help of Figs. 6 and  7, we can choose the strategy to suppress wild mosquitoes based on the actual conditions and requirement.

Recently, by releasing *Wolbachia*-infected mosquitoes twice a week for three years (Zheng et al. 2019), our team shows that combining incompatible and sterile insect techniques (IIT-SIT) enables near elimination of the populations of *Aedes albopictus* in Shazai island, Guangzhou. To prevent bites from the female mosquitoes mixed in the released males, measures should be taken to reduce or even eliminate the number of females in each release. For the case that all the subsequent released mosquitoes are male, we can consider sex structure in (4) by changing *R*(*t*) to 2*R*(*t*), and the related discussions are nearly the same. We believe that the combination of spraying pesticides and releasing *Wolbachia*-infected mosquitoes can play an important role in mosquito control. In this work, we studied the mosquito replacement in a normal environmental condition and high temperature condition with heatwaves, respectively. However, the actual habitat conditions are changeable and difficult to be described by two simple stable conditions. Furthermore, the mosquito population can be also affected by many other factors, such as migration (Schmidt and Barton 2017) and urbanization process (Li and Kamara 2014). The specific effects of insecticides are also complicated. In the future work, we will explore more information of mosquito growth and make good use of the advantages of the two measures to formulate mathematical models to study mosquito control.
